# Patterns of Retinal Damage Facilitate Differential Diagnosis between Susac Syndrome and MS

**DOI:** 10.1371/journal.pone.0038741

**Published:** 2012-06-11

**Authors:** Alexander U. Brandt, Hanna Zimmermann, Falko Kaufhold, Julia Promesberger, Sven Schippling, David Finis, Orhan Aktas, Christian Geis, Marius Ringelstein, E. Bernd Ringelstein, Hans-Peter Hartung, Friedemann Paul, Ilka Kleffner, Jan Dörr

**Affiliations:** 1 NeuroCure Clinical Research Center, Charité – Universitätsmedizin Berlin, Berlin, Germany; 2 Department of Ophthalmology, University of Münster, Münster, Germany; 3 Institute for Neuroimmunology and Clinical Multiple Sclerosis Research (inims), University Medical Center Hamburg Eppendorf, Hamburg, Germany; 4 Department of Ophthalmology, University Medical Center, Heinrich-Heine-University, Düsseldorf, Germany; 5 Department of Neurology, University Medical Center, Heinrich-Heine-University, Düsseldorf, Germany; 6 Department of Neurology, University of Würzburg, Würzburg, Germany; 7 Department of Neurology, University of Münster, Münster, Germany; 8 Clinical and Experimental Research Center for Multiple Sclerosis, Department of Neurology, Charité – Universitätsmedizin Berlin, Berlin, Germany; 9 Experimental and Clinical Research Center, Max Delbrück Center for Molecular Medicine and Charité – Universitätsmedizin Berlin, Berlin, Germany; Charité Universitätsmedizin Berlin, NeuroCure Clinical Research Center, Germany

## Abstract

Susac syndrome, a rare but probably underdiagnosed combination of encephalopathy, hearing loss, and visual deficits due to branch retinal artery occlusion of unknown aetiology has to be considered as differential diagnosis in various conditions. Particularly, differentiation from multiple sclerosis is often challenging since both clinical presentation and diagnostic findings may overlap. Optical coherence tomography is a powerful and easy to perform diagnostic tool to analyse the morphological integrity of retinal structures and is increasingly established to depict characteristic patterns of retinal pathology in multiple sclerosis. Against this background we hypothesised that differential patterns of retinal pathology facilitate a reliable differentiation between Susac syndrome and multiple sclerosis. In this multicenter cross-sectional observational study optical coherence tomography was performed in nine patients with a definite diagnosis of Susac syndrome. Data were compared with age-, sex-, and disease duration-matched relapsing remitting multiple sclerosis patients with and without a history of optic neuritis, and with healthy controls. Using generalised estimating equation models, Susac patients showed a significant reduction in either or both retinal nerve fibre layer thickness and total macular volume in comparison to both healthy controls and relapsing remitting multiple sclerosis patients. However, in contrast to the multiple sclerosis patients this reduction was not distributed over the entire scanning area but showed a distinct sectorial loss especially in the macular measurements. We therefore conclude that patients with Susac syndrome show distinct abnormalities in optical coherence tomography in comparison to multiple sclerosis patients. These findings recommend optical coherence tomography as a promising tool for differentiating Susac syndrome from MS.

## Introduction

Susac syndrome is a rare disease characterised by the clinical triad of encephalopathy, vision disturbances, namely visual field defects, and sensorineural hearing loss [1]–[3]. The exact prevalence of Susac syndrome is unknown, and its pathogenesis is still unclear; autoimmune processes that lead to an occlusion of small vessels in the brain, retina and inner ear are believed to play an important role [4], [5]. The disease most often manifests in the third to fourth decade [6]. The prognosis mainly depends on the severity, the often self-limited and monophasic, sometimes fluctuating and rarely relapsing clinical course [7], and the appropriate treatment [8], [9]. Retinal infarction presenting with scotoma is one of the clinical hallmarks, although often not predominant. Patients can present with episodic or permanent vision loss [6]. Fluorescein angiography (FAG) and funduscopy show branch retinal artery occlusions (BRAO), arterial wall hyperfluorescence and retinal arterial wall plaques, termed Gass plaques [10]. Retinal involvement can be missed in cases where patients do not complain about visual disturbances due to neuropsychological impairment, or when physicians are not familiar with the disease. In fact, in several reported cases BRAO was only detected after repeated FAG [6].

The diagnosis of Susac syndrome is straightforward, when the characteristic clinical triad is complete, when the physician is familiar with the clinical presentation, and when the crucial diagnostic procedures are carried out and show characteristic findings like BRAO in FAG. However, the diagnosis is often complicated by the fact that the characteristic signs usually do not occur concomitantly but rather develop successively with symptom-free intervals [3], which often enough results in a delayed or even completely missed diagnosis. Consensus criteria for the diagnosis of Susac syndrome have not yet been established. In MRI, Susac syndrome usually presents with “punched-out” lesions, frequently in the corpus callosum and periventricular area [11]. A number of differential diagnoses, most of which occur more frequently than Susac syndrome, have to be taken into consideration [3]. In turn, Susac syndrome should be considered as differential diagnosis in various conditions. Due to some overlap in the clinical presentation and the patterns of MRI pathology multiple sclerosis (MS) is probably the most frequent misdiagnosis of Susac syndrome [2], [8], [11], [12]. However, with respect to the different therapeutic approach, particularly the necessity of a first-line immunosuppressive treatment in Susac syndrome in contrast to primarily immunomodulatory approaches in MS, a prompt establishment of the diagnosis is essential. Additional diagnostic criteria allowing an early differential diagnosis are therefore highly warranted.

Optical coherence tomography (OCT) has recently become a valuable addition to the neurologist's diagnostic toolbox, proving its usefulness in a variety of disorders with neuro-ophthalmologic involvement [13]–[16]. OCT facilitates e.g. non-invasive quantification of both the thickness of the retinal nerve fibre layer (RNFLT), which represents unmyelinated axons of retinal ganglia converging to the optic disc to form the optic nerve and the macular volume which represents the volume of the central retina [13]–[16]. In a recently published case report we could demonstrate pathologic OCT findings in a patient with Susac syndrome [3]. Based on these findings we hypothesised that retinal changes (i) can be regularly detected by OCT in patients with Susac syndrome and (ii) differ from retinal pathology observed in MS. A different pattern of retinal pathology in Susac syndrome and MS would be of clinical value in terms of distinguishing patients with Susac syndrome from MS patients.

## Methods

### Objectives

To identify OCT changes in patients with Susac syndrome and to compare these changes with matched healthy controls and multiple sclerosis patients.

### Study Design and Participants

This is a prospective, cross-sectional, multicentre observational study documenting OCT findings in Susac syndrome patients. Patients with definite diagnosis of Susac syndrome, aged ≥ 18 years were recruited from the neurologic outpatient clinics of five large university medical centres (Berlin, Münster, Düsseldorf, Hamburg, and Würzburg, Germany). Exclusion criteria were inability to provide informed consent. All included patients underwent complete neurological examination. Medical history, particularly with respect to encephalopathy, visual symptoms, and hearing loss was taken from all study participants. In cases where the classical clinical triad was not present, diagnosis was established on clinical presentation and MRI findings. Visual testing was performed with bedside visual field testing. Age and gender matched healthy controls (HC) and patients with relapsing remitting MS (RRMS) were randomly selected from the imaging research database of the NeuroCure Clinical Research Center (NCRC) at Charité – Universitätsmedizin Berlin by an investigator blinded to the OCT data.

### Ethics

The study was approved by the local ethics committees and was conducted in accordance to the Declaration of Helsinki in its currently applicable version, the guidelines of the International Conference on Harmonisation of Good Clinical Practice (ICH-GCP), and the applicable German laws. All participants gave informed written consent.

### Optical Coherence Tomography

RNFLT and TMV were measured with Stratus 3000 OCT (Carl Zeiss Meditec, California, USA) using “Fast RNFL 3.4” and “Fast Macula Thickness Map” protocols (software V4.0) by trained personnel. For RNFLT, a 3.4 mm diameter circular scan was acquired circumferentially to the optic disc, and for TMV six radial lines were taken, centred within the fovea. A good quality image was defined as having generalised signal distribution, a reflectance signal from either RNFL or retinal pigment epithelium strong enough to identify either layer, no missing parts caused by eye movements, and a signal strength of ≥7 of 10 [17]. Segmentation lines for upper and lower borders of RNFL were required to be on the internal limiting membrane and lower border of the RNFL. For the comparison of OCT measurements to normative data, the device's internal normative database, comprising of measurements of 170 eyes from HC was used as a reference. Percentile positions of measurements compared to these normative data are automatically given on the device's TMV and RNFLT report as below 1^st^ percentile, between 1^st^ and 5^th^ percentile, between 5^th^ and 95^th^ percentile, and above 95^th^ percentile.

### Statistical methods

Differences in age and time since diagnosis between patients with Susac syndrome, HC and RRMS patients with and without a history of optic neuritis were analysed using Friedman's analysis for matched pairs. Differences between eyes from the groups were assessed using generalised estimating equation models (GEE) accounting for intra-patient/inter-eye dependencies. To further rule out possible age related effects or effects related to minor differences in time since diagnosis, GEE models were corrected for age and additionally with time since diagnosis for comparisons against RRMS patients. In all GEE, the diagnostic group was used as independent categorical variable. Mean values in text are given with standard deviation (SD) after a ± sign. All statistical tests were performed using SPSS 20 (IBM, Somers, NY, USA). For all calculations, statistical significance was established at p<0.05.

## Results

### Cohort description

Nine patients with Susac syndrome were prospectively recruited (six jointly from Berlin and Münster, one from each of the other three centres). A demographic overview is given in table 1, and a multiple case presentation is provided in table 2. The female to male ratio of 2∶1 reflected that reported in the literature. All patients had a history of hearing loss, and all but one patient (P2) had symptoms of encephalopathy. All patients except one (P6) had a history of visual symptoms and provided accompanying reports of BRAO. Additionally, one patient (P2) had visual symptoms on the right eye. However, in this patient a report of BRAO did exist for both eyes. At the time of OCT investigation, no patient was in a clinically active phase of the disease.

**Table 1:** Demographic overview of Susac patients included in the study.

Subjects	n	9
Eyes	n	18
Gender	Male (%)	3 (33)
	Female (%)	6 (67)
Age (years)	Mean ± SD	33±11
	Min – Max	20–47
Time since diagnosis (months)	Mean ± SD	65±55
	Min – Max	3–173
Encephalopathy	No (%)	1 (11)
	Yes (%)	8 (89)
Hearing loss	No (%)	0 (0)
	Yes (%)	9 (100)
Visual impairment (eyes)	No (%)	3 (17)
	Yes (%)	15 (83)
	No (%)	3 (17)
	Yes (%)	15 (83)

*Abbreviations:* BRAO  =  branch retinal artery occlusion, SD  =  standard deviation.

**Table 2:** Optical coherence tomography data of the nine patients with Susac syndrome.

Pat.	Sex	Age	Dur.	Eye	VS	BRAO	TMV [mm^3^]	A [µm]	T [µm]	S [µm]	N [µm]	I [µm]
**P1**	m	20	26	OD	yes	yes	6.00	85	48	101	70	122
				OS	yes	yes	7.22	83	65	107	47	115
**P2**	m	32	58	OD	yes	yes	7.64	105	81	132	106	102
				OS	no	yes	7.66	108	77	141	98	115
**P3**	f	44	173	OD	yes	yes	5.67	61	51	54	51	88
				OS	yes	yes	6.11	61	49	64	53	77
**P4**	m	39	50	OD	yes	no	5.70	60	53	78	49	59
				OS	yes	yes	6.63	82	63	95	60	111
**P5**	f	22	41	OD	yes	yes	6.26	67	65	58	64	83
				OS	yes	yes	6.21	73	67	69	67	89
**P6**	f	30	128	OD	no	no	6.61	88	76	84	71	121
				OS	no	no	6.64	93	70	125	64	115
**P7**	f	20	66	OD	yes	yes	6.52	96	74	103	74	131
				OS	yes	yes	6.86	115	72	132	107	149
**P8**	f	45	69	OD	yes	yes	5.25	62	52	70	41	83
				OS	yes	yes	5.55	52	57	56	31	65
**P9**	f	47	3	OD	yes	yes	6.66	86	74	103	63	103
				OS	yes	yes	6.57	79	69	99	56	93

*Abbreviations:* Pat.  =  Patient No; Age  =  age of onset; Dur.  =  time since diagnosis at time of OCT measurement in months; OD  =  right eye; OS  =  left eye; VS  =  visual symptoms; BRAO  =  branch retinal artery occlusion; TMV  =  total macular volume in mm^3^; A  =  average retinal nerve fibre layer thickness (RNFLT) in µm; T  =  temporal, S  =  superior, N  =  nasal, I  =  inferior quadrant's RNFLT in µm.

### Macular and retinal nerve fibre layer damage

**Figure 1:**
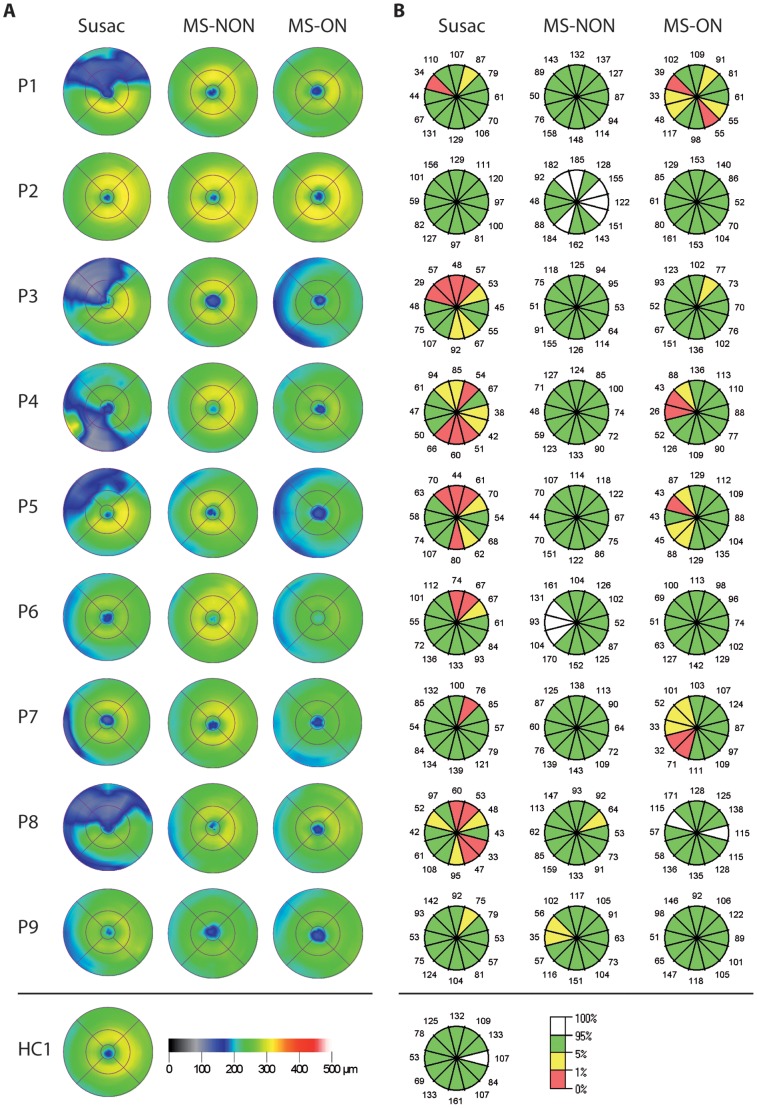
Macular and ring scans from patients with Susac syndrome and matched RRMS patients. Shown are only the left eyes (randomly selected to save space) from each Susac patient (P1-9) and the corresponding left eyes from RRMS patients without history of optic neuritis (MS-NON) and RRMS patients with history of optic neuritis (MS-ON). On the bottom, a comparison of scans from one of the healthy controls is given. A) Colour coded is the calculated macular thickness from the device's segmentation algorithm with black to blue for reduced thickness and yellow to green for normal thickness (left legend on the bottom). The macular thickness map is calculated from six linear scans through the centre of the macula. Of note is the different distribution of the damage. B) For RNFLT scans, the thickness from 12 clock-hour segments of the circular scan is given. Colour coded is the thickness relative to the normative database with green and white meaning normal values above the 5^th^ or 95^th^ percentile and yellow and red meaning reduction of thickness below the 5^th^ or 1^st^ percentile (right legend on the bottom). Whereas some Susac patients' eyes show striking sectoral damage, eyes from RRMS patients show an even thinning with an accentuation in the outer temporal areas, that is further pronounced with a history of optic neuritis. Three Susac patients (P6, 7, 9) show a similar pattern.

**Figure 2:**
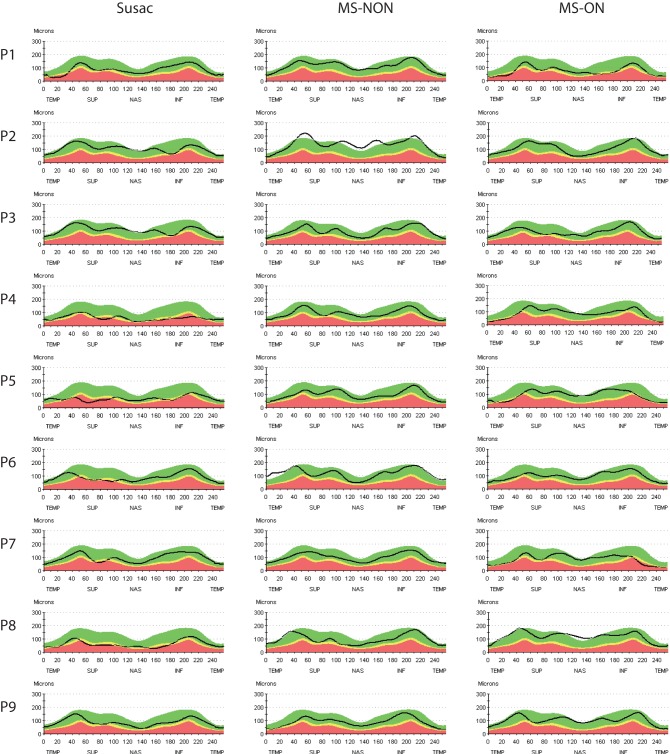
RNFLT from patients with Susac syndrome and matched RRMS patients. Shown are only the left eyes (randomly selected to save space) from each Susac patient (P1-9) and the corresponding left eyes from RRMS patients without history of optic neuritis (MS-NON) and RRMS patients with history of optic neuritis (MS-ON). Each graph represents the RNFLT from a peripapillary ring scan. Colour coded is the thickness relative to the normative database with green and white meaning normal values above the 5^th^ or 95^th^ percentile and yellow and red meaning reduction of thickness below the 5^th^ or 1^st^ percentile. Abbreviations: RNFLT  =  retinal nerve fibre layer thickness.

Single case OCT measurements are given in table 2, and a synopsis is provided in table 3. In summary, compared to the normative database of the OCT device, most patients showed a reduction in either average RNFLT or macular measurements. All but one patient (P2) with a history of visual symptoms showed either reduced average RNFLT and/or diminished TMV in at least one eye. P2 showed a very mild clinical phenotype and TMV was above the 95^th^ percentile when compared to the normative database of the device. Interestingly, one patient without visual symptoms and without documented BRAO (P6) did also present a pathological OCT with a strong RNFLT reduction in the superior quadrant of the right eye, although average RNFLT in this eye was normal (figures 1 and 2).

**Table 3:** Mean values from optical coherence tomography measurements of the macula (total macular volume and below) and the circular scan around the optic nerve head (RNFLT Average and below).

		Susac				HC				MS-NON				MS-ON			
		Mean	SD	Min	Max	Mean	SD	Min	Max	Mean	SD	Min	Max	Mean	SD	Min	Max
**Total Macular Volume [mm^3^]**		**6,43**	**0,67**	**5,25**	**7,66**	**7,14**	**0,35**	**6,65**	**7,84**	**7,09**	**0,36**	**6,51**	**7,75**	**6,73**	**0,49**	**6,04**	**7,68**
**Inner Macula**	**T [µm]**	240	33	172	284	269	18	238	296	269	13	237	286	253	20	220	294
	**S [µm]**	242	55	128	300	283	16	255	308	281	17	239	301	265	19	237	296
	**N [µm]**	271	26	207	306	282	17	256	312	279	16	243	302	266	19	241	302
	**I [µm]**	254	42	146	301	280	18	254	311	281	12	258	301	260	21	232	304
**Outer Macula**	**T [µm]**	205	27	160	243	229	11	213	252	229	11	211	251	219	18	193	252
	**S [µm]**	209	42	139	263	247	12	231	276	245	14	222	266	234	17	209	263
	**N [µm]**	246	18	211	267	264	14	248	291	262	16	232	291	248	18	214	281
	**I [µm]**	222	30	143	265	242	12	223	262	241	11	216	267	226	18	200	266
**RNFLT Average [µm]**		**81**	**18**	**52**	**115**	**107**	**9**	**91**	**120**	**102**	**14**	**87**	**137**	**95**	**11**	**74**	**118**
**RNFLT**	**T [µm]**	65	11	48	81	75	12	59	101	73	17	49	109	57	14	29	77
	**S [µm]**	93	28	54	141	133	14	104	158	123	17	104	164	113	16	87	150
	**N [µm]**	65	21	31	107	85	16	67	120	82	21	47	142	90	16	62	123
	**I [µm]**	101	24	59	149	132	14	109	150	128	17	105	163	120	16	89	139

*Abbreviations:* RRMS  =  relapsing-remitting multiple sclerosis; HC  =  healthy controls; SD  =  standard deviation; RNFLT  =  retinal nerve fibre layer thickness; t  =  temporal; S  =  superior; N  =  nasal; I  =  inferior.

Importantly, TMV and RNFLT reduction were not evenly distributed over the entire scanning area but scattered over all sectors (figure 1 and figure 2). Patients with Susac syndrome showed a RNFLT below the 5^th^ percentile in a mean 3.6±3.4 (range 0–10) of the twelve clock-hour sectors and a TMV below the 5^th^ percentile in a mean 2.4±2.9 (range 0–7) of the nine macular sectors whereas the other sectors were within normal ranges. All but one patient (P2) showed this patchy retinal damage in at least one eye as indicated by the yellow and red areas in the RNFLT measurements and the dark blue areas in the TMV measurements (figure 1).

### Comparison to matched healthy controls and multiple sclerosis patients

To compare OCT results from patients with Susac syndrome to results from HC and RRMS patients, either group of nine gender and age matched HC (mean age 33±11 years, 3/6 male/female), nine RRMS patients without any history of optic neuritis (mean age 32±10 years, 3/6 male/female, time since diagnosis 55±40 months) and nine RRMS patient with a previous bilateral optic neuritis (mean age 33±9 years, 3/6 male female, time since diagnosis 38±40 months) was considered. HC and RRMS patients were matched for gender, age and time since diagnosis. Gender was matched exactly 1∶1. The differences in age were not significant (Friedman's analysis for matched pairs p = 0.661). Likewise, time since diagnosis of RRMS patients with and without previous optic neuritis was statistically not different from Susac patients (Friedman's analysis for matched pairs p = 0.062).

**Figure 3:**
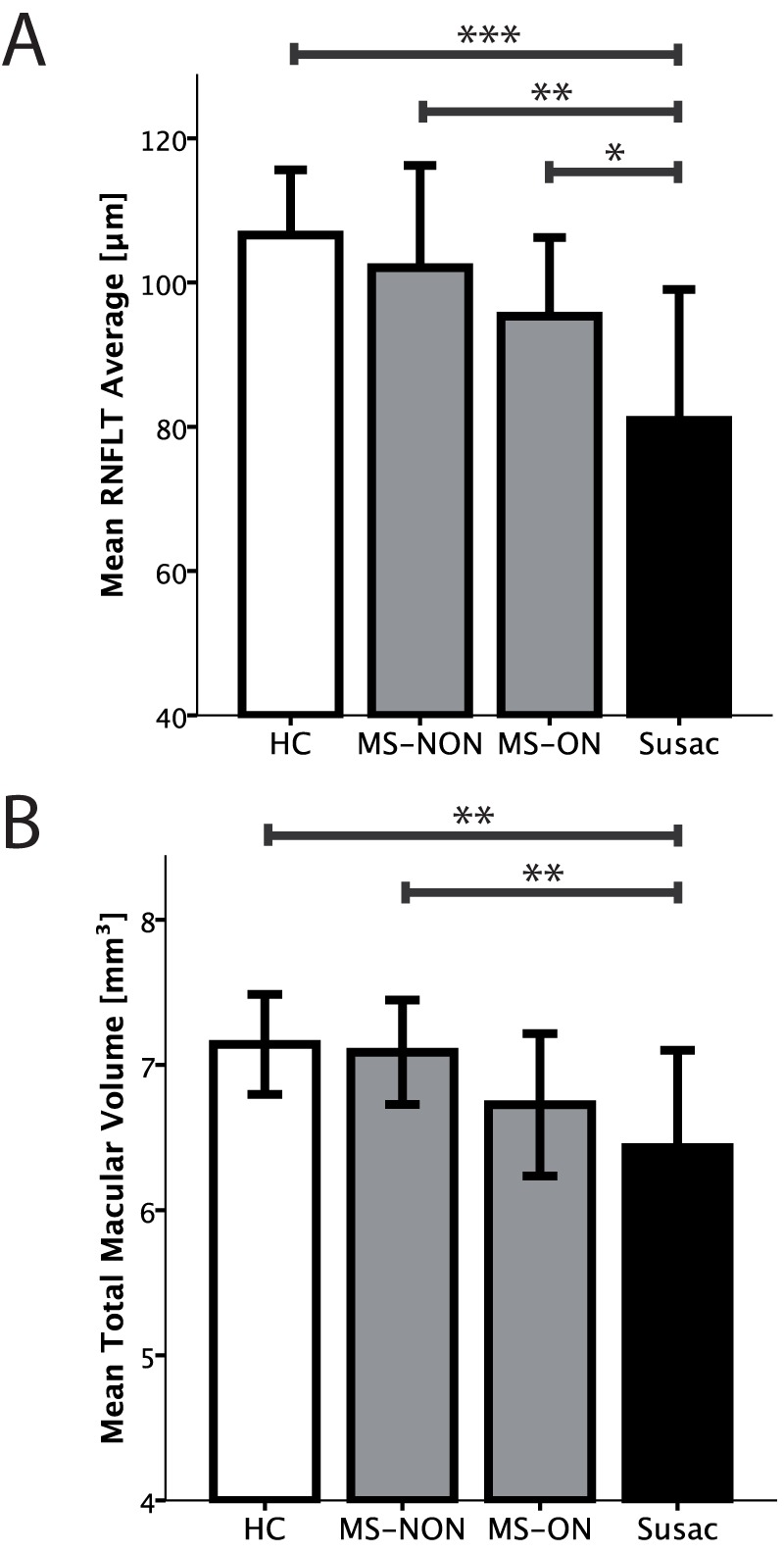
Comparison between healthy controls, RRMS patients and patients with Susac syndrome. Error bars indicate 1-fold standard deviation. Significance level from Generalised Estimating Equations are given as ***) p<0.001, **) p<0.01, and *) p<0.05. *Abbreviations*: HC  =  healthy control; RRMS  =  relapsing remitting multiple sclerosis; RNFLT  =  retinal nerve fibre layer thickness; MS-ON  =  RRMS patients with a previous, bilateral optic neuritis; MS-NON  =  RRMS patients without a history of optic neuritis.

Average RNFLT was reduced in patients with Susac syndrome (average RNFLT 81±18 µm, table 3) in comparison to HC (average RNFLT 107±9 µm, coefficient B = −25.5, SE 6.2, p<0.001, GEE), RRMS patients without previous optic neuritis (average RNFLT 102±14 µm, coefficient B = −21.3, SE 6.2, p = 0.001, GEE) and RRMS patients with a history of optic neuritis (average RNFLT 95±11 µm, coefficient B = −13.5, SE 5.8, p = 0.019, GEE) (figure 1B, 2 and 3). Accordingly, TMV was reduced in patients with Susac syndrome (TMV 6.43±0.67 mm^3^, table 3) in comparison to HC (TMV 7.14±0.35 mm^3^, coefficient B = −0.71, SE 0.22, p = 0.001, GEE), RRMS patients without history of optic neuritis (TMV 7.09±0.36 mm^3^, coefficient B = −0.67, SE 0.20, p = 0.001, GEE) but not against RRMS patients with previous history of optic neuritis (TMV 6.73±0.49 mm^3^, coefficient B = −0.23, SE 0.21, p = 0.224, GEE) (figure 1 and 3).

## Discussion

In this cross-sectional, observational study we investigated nine patients with Susac syndrome using OCT and compared RNFLT and TMV data with nine gender and age matched HC and nine RRMS patients each with or without history of optic neuritis. Our main findings are (a) pathologic OCT measurements in most of the Susac patients with a history of visual symptoms when compared to the normative database of the OCT device; (b) reduced RNFLT and macular measurements when compared to gender and age matched HC; (c) a more severe retinal nerve fibre damage in patients with Susac syndrome as compared to RRMS patients irrespective the history of optic neuritis despite a similar disease duration, and most importantly (d) distinct patterns of retinal or retinal nerve fibre layer damage among patients with Susac syndrome compared to RRMS patients that can help to discriminate between both diseases.

Although a rare disease, Susac syndrome needs to be considered in the differential diagnosis of a variety of neurological disorders. Currently, its diagnosis is based primarily on the clinical presentation, the documentation of BRAO by FAG, and characteristic findings on cranial magnetic resonance imaging, including subtle changes such as fibre impairment detected by diffusion tensor imaging [18]. Recently, anti-endothelial antibodies in Susac syndrome were reported as a potential future diagnostic criterion and as a possible pathologic correlate [19], [20].

The majority of patients with Susac syndrome in this study showed a characteristic and thus very distinct pattern of often severe and patchy retinal nerve fibre thinning in RNFLT and retinal damage in TMV. Compatible with the pathology of this retinal microangiopathy, retinal damage was usually scattered over distinct foci and not evenly distributed: whereas several sectors in RNFLT and/or TMV showed severe damage, other sectors remained completely normal. The notable exclusion from that rule was patient P2, who had a mostly normal RNFLT and TMV, despite visual symptoms and BRAO findings in FAG.

This presentation of Susac syndrome in OCT is in contrast to OCT findings in MS. Several publications that were recently reviewed in a meta-analysis [16], report an evenly distributed thinning of RNFL in MS that is slightly enhanced on the temporal quadrant after optic neuritis [21]. However, RNFL thinning in MS accumulates over time and becomes more severe especially in later stages of the disease. In early stages of MS and in clinically isolated syndrome (CIS), when differentiation of Susac syndrome is most important, RNFL thinning is barely detectable [22]. However, since Susac patients in this study's cohort had an established diagnosis already for several months or even years, it is not clear, how early Susac syndrome with acute visual impairment and BRAO translate into pathological OCT findings.

The crucial question in this context is at what time point OCT starts to show abnormal findings in patients with Susac syndrome. A few case reports and one study with nine BRAO patients without underlying Susac syndrome report an initial thickening of RNFL leading to final thinning after several months [23]–[25]. In general, one would suspect that structural retinal nerve fibre damage evolves some time after the underlying vessel pathology. One patient (P6) however showed an abnormal OCT even without BRAO in FAG pointing towards a potential usability of OCT in earlier stages of the disease when the diagnosis is yet not fully established. This issue should be addressed in a longitudinal study investigating the development of retinal lesions of newly diagnosed patients with Susac syndrome and BRAO over time including functional visual outcomes. Due to the design of our study, it was not possible to perform FAG and OCT at the same time, unfortunately limiting the possibility to make assertions on the co-occurrence of OCT and BRAO findings in this respect. Therefore, these questions are currently investigated in a follow up study.

Beyond differential diagnosis, the severe structural retinal damage in Susac syndrome especially in the macular scans detected by OCT supports an aggressive treatment regimen early after diagnosis [8]. This notion is further assisted by the fact that patient (P6) without visual symptoms showed a pathological OCT, suggesting early subclinical retinal damage. On the other hand, one patient (P2) with actual BRAO did not have pathological findings in OCT. Thus, BRAO does not always lead to pathological OCT findings. The dissociation of retinal damage and history of BRAO observed in P2 and P6 might suggest additional mechanisms independent of BRAO underlying retinal damage in this disease.

The reported study has important limitations that need to be considered. Due to the rarity of the disease, recruitment of a sufficient number of patients is challenging and prospective data on Susac syndrome derived from studies with more than five patients hardly exist. Although data on a limited number of nine patients obviously need to be interpreted cautiously, our study is among the largest prospective studies so far reported on Susac syndrome [26]–[28]. However, conclusions on the discriminatory ability of OCT between Susac syndrome and MS should be interpreted with care from this study with only small numbers.

Another important limitation of our study is the use of a time domain OCT device that measures macular volume using a six-line scan protocol instead of a volume/3D scan. A thickness map is generated via interpolating the measurements between the six line scans. The distinct and striking sectorial damage in the macular scans might therefore be under- or overestimated. Since the macular scan incorporates all retinal layers between the inner limiting membrane and the retinal pigment epithelium, it is not possible to determine via time domain OCT alone, which retinal layers are affected in Susac syndrome in comparison to optic neuritis. Because of the vascular nature of the disease, one might speculate though, that any damage would be more profound and affecting more retinal layers when compared to optic neuritis. Furthermore, the used time domain OCT device has known limitations in the reproducibility of sectoral RNFLT [29]. Next generation spectral domain OCT devices provide volume 3D scans and intra-retinal segmentation algorithms [30], possibly further enhancing the value of OCT in the differential diagnosis of Susac syndrome. However, in contrast to spectral domain OCT, time domain OCT is already widely available and patients with Susac syndrome show the reported distinct phenotype in comparison to MS even in time domain OCT, thus strengthening the importance of OCT application in differential diagnosis in routine or outpatient clinic settings.

In summary, we show that Susac patients regularly have distinct abnormalities in OCT scans. The sectorial pattern of retinal damage supports the hypothesis of a vascular origin with patchy lesions. Most importantly, our data recommends OCT as a tool in early primary and secondary diagnostics of Susac syndrome when differentiation from MS and other neuroimmunologic diseases can prove challenging.
